# Organic amendments and conservation tillage improve cotton productivity and soil health indices under arid climate

**DOI:** 10.1038/s41598-022-18157-0

**Published:** 2022-08-18

**Authors:** Saeed Ahmad, Ijaz Hussain, Abdul Ghaffar, Muhammad Habib ur Rahman, Muhammad Zain Saleem, Muhammad Waqas Yonas, Hammad Hussnain, Rao Muhammad Ikram, Muhammad Arslan

**Affiliations:** 1 Department of Agronomy, MNS-University of Agriculture Multan-Pakistan, Punjab, 66000 Pakistan; 2 Crop Science Group, Institute of Crop Science and Resource Conservation (INRES), University Bonn, Bonn, Germany; 3 College of Agriculture, Bahadur Sub Campus, Bahauddin Zakariya University, Layyah, 31200 Pakistan; 4 College of Resources and Environment, Southwest University, Tiansheng Road Beibei, Chongqing, 400715 People’s Republic of China; 5 Cotton Research Institute Multan, Ayub Agriculture Research Institute, Faisalabad, Pakistan

**Keywords:** Plant sciences, Environmental sciences

## Abstract

Long-term different tillage system field trials can provide vital knowledge about sustainable changes in soil health indices and crop productivity. This study examined cotton productivity and soil health indices under different tillage systems and organic materials. The present study was carried out at MNS University of Agriculture, Multan to explore the effect of different tillage systems: conventional tillage (T_1_), conservation tillage (T_2_), and organic materials: control (recommended dose of synthetic fertilizers; 160:90:60 kg ha^−1^NPK), poultry manure (10 t ha^−1^ PM), compost (10 t ha^−1^ CM), farmyard manure (20 t ha^−1^ FYM), and biochar (7 t ha^−1^ BC) on cotton productivity and soil health indices. Two years field trials showed that different tillage systems and organic materials significantly improved the growth, morphological, and yield attributes of cotton and soil health indices. The cotton showed highest seed cotton yield (3692–3736 kg ha^−1^), and soil organic matter (0.809–0.815%), soil available nitrogen (74.3–74.6 mg kg^−1^), phosphorus (7.29–7.43 mg kg^−1^), and potassium (213–216 mg kg^−1^) under T_2_ in comparison to T_1_ system during both years of field experiment, respectively. Similarly, PM (10 t ha^−1^) showed highest seed cotton yield (3888–3933 kg ha^−1^), and soil organic matter (0.794–0.797%), nitrogen (74.7–75.0 mg kg^−1^), phosphorus (7.39–7.55 mg kg^−1^), and potassium (221–223 mg kg^−1^) when these are compared to FYM (20 t ha^−1^), CM (10 t ha^−1^), and BC (7 t ha^−1^) during both years of field experiment, respectively. These findings indicate that conservation tillage system with application of 10 t ha^−1^ PM are the best practices for the sustainable cotton production and to ensure improvement in the soil health indices under arid climatic conditions.

## Introduction

The continuous and excessive application of synthetic fertilizers leads to harmful effects on soil, plants, animals and humans health^1^. Despite of these adverse effects, chemical fertilizers especially nitrogenous fertilizers are being extensively used in agriculture sector^2^. In present cotton production system, nitrogenous fertilizers are being used extensively leading to higher nitrogen losses by leaching away from the soil and enhanced environmental pollution^3,4^. Furthermore, the improper and continuous use of synthetic fertilizers lead to soil degradation, reduced water holding capacity (WHC) of soil, increased soil erosion and losses of soil nutrients ultimately soil fertility which are vital concerns being faced by agriculture lands worldwide^5^. To cope with these problems, application of organic materials such as biochar, farmyard manure and poultry manure as a part of integrated nutrient management strategy is considered as the vital and sustainable approach to sustain and enhance soil health, and crop productivity^6,7^.

Compost is a great and sustainable option of organic farming because it reduces the leaching losses of nutrients, and minimizes dependence on synthetic fertilizers^8,9^. It is also an eco-friendly source of micro (Zn, Fe, Cu, and Mn) and macro (N, P, K, Ca, and Mg) nutrients depending on the composition and nature of composted waste material^10^. Furthermore, compost is reliable option to improve soil physical^11^, chemical^12^, and biological^13^ traits in agricultural lands. Additionally, compost application also increases the crop productivity which is attributed to its imperative role in improving soil fertility and provision of micro and macro nutrients^14,15^. Several studies have demonstrated positive effects of compost on growth, and morphological attributes of cotton^9,16^. Biochar is also considered as an eco-friendly alternative to synthetic fertilizers produced through the thermo-chemical decomposition of plants residues and organic wastes^1,6^. It is a brilliant source of stable carbon which sustains and improves water holding capacity, soil microbial activity, soil porosity, and nutrient and water retention capacity ultimately soil fertility and crop productivity^5,17,18^. Additionally, long-term application of biochar also increases the availability of N, P, K, and soil organic matter^6^. Different field research trials have showed that cotton crop produced higher growth, morphological, and yield traits with the biochar application under arid climatic conditions^6,7^. In addition, it has become a viable option to get higher crop productivity from the highly degraded or weathered soils as it decreases nitrate leaching and ammonia volatilization losses from such kind of soils^19,20^.

Poultry manure is an excellent source of organic fertilizers which consists of large proportion of macro (N, P, and K) and micro (Zn, Fe, Mn, Mn, and Cu) nutrients^6,21^. It improves the physical attributes of soil, nutrients uptake and ultimately crop productivity^22,23^. Hence, soil incorporation of poultry manure results in higher nutrients uptake of N, P and K as well as higher nitrogen use efficiency in cotton production^24,25^. Higher cotton yields have been recorded with poultry manure application as compared to cotton yield obtained by the 100% application of synthetic fertilizers^26,27^. Furthermore, one investigation has showed that poultry manure application in combination with half of recommended dose of synthetic fertilizers produced higher growth, morphological and yield traits in comparison to recommended dose of synthetic fertilizers^6^. Higher productivity of field crops with poultry manure application is attributed to its specific role in improving soil structure, reducing soil compaction and retention of maximum soil water content^28,29^. Moreover, farmyard manure is considered a hub of macronutrients (N, P, K) and to some extent micro-nutrients (Fe, Mn and Zn) ^30,31^. Farmyard manure improves soil health, nutrients availability in soil and higher cotton crop productivity^28^. Hence, soil incorporation of farmyard manure reduces soil bulk density, improves soil porosity, soil structure, reduces the soil compaction and soil degradation which leads to enhanced cotton crop productivity by improving nitrogen use efficiency (NUE) and nutrients uptake^32,33^. Moreover, soil incorporation of farmyard manure in combination with half dose of synthetic fertilizers produced higher growth, morphological and yield traits in comparison to recommended dose of synthetic fertilizers^6^.

Minimum tillage is one of the major conservation tillage operations that have been widely used for improving soil health indices and minimizing the adverse environmental impacts of intensive farming practices^34^. The adaptation of conservation tillage systems especially minimum tillage in combination with organic manures may sustain the soil environment to improve crop growth and cotton crop productivity in long run by reducing soil erosion and carbon emissions^35,36^. The minimum tillage systems improves soil health indicators (organic matter, N, P, and K), reduces soil degradation, enhanced moisture accessibility for crops, necessary for higher cotton crop productivity^34,37,38^. Many studies have reported that the improved results for soil health indicators (soil organic matter, N, P, and K) and crop parameters with the application of organic manures in combination with minimum tillage systems^14,39^.

There are so many research works executed on the effects of organic amendments and inorganic fertilizers as well as different systems on soil properties, growth, and yield of crops worldwide. However, no extensive research work has been conducted to evaluate combined effects of tillage systems and application of organic amendments especially biochar, compost, poultry and farmyard manure on soil health indices and cotton productivity under arid climatic conditions. For this purpose, we hypothesized that the conservation tillage and soil incorporation with different organic materials may improve soil health indices, growth, morphological traits and ultimately cotton crop productivity. Hence, the present study focused on the influence of application of biochar, compost, and farmyard and poultry manures on the soil health indices, growth, morphological and yield related traits of cotton crop under different tillage systems.

## Results

### Growth attributes and chlorophyll content of cotton crop

**Figure 1:**
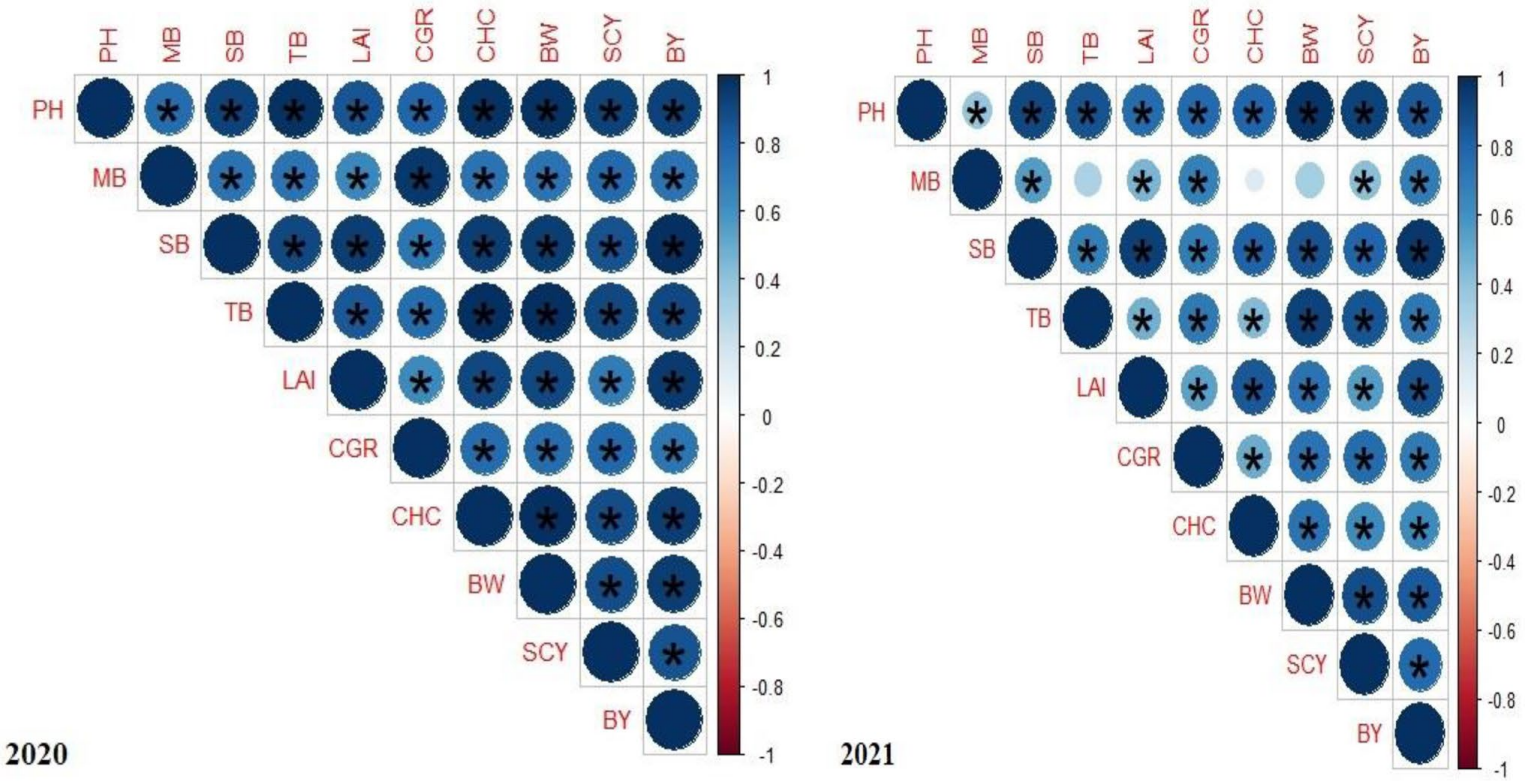
Correlation map showing the effect of different tillage systems, and organic materials on different traits (growth, chlorophyll content, morphological, and yield) of cotton. The areas of circles show the absolute value of corresponding correlation coefficients tested at *0.01 significance level. *BY* biological yield, *SCY* seed cotton yield, *BW* boll weight, *CHC* chlorophyll content, *CGR* crop growth rate, *LAI* peak leaf area index, *TB* total bolls, *SB* sympodial branches, *MB* monopodial branches, *PH* plant height. *p ≤ 0.05; **≤ 0.01; ***≤ 0.001.

 Crop growth rate, peak leaf area index, and chlorophyll content of cotton crop were markedly affected (at p ≤ 0.05) by different tillage systems, and organic materials. However, the interactive effects of different tillage systems, and organic materials, year as source of variation and its interaction with different tillage systems and organic materials were not found significant for the crop growth rate, peak leaf area index, and chlorophyll content of cotton crop (Table 1). Cotton crop sown by conservation tillage system showed significantly higher crop growth rate, peak leaf area index, and chlorophyll content as compared with conventional tillage during both years of the field experimentation. Furthermore, crop growth rate, peak leaf area index, and chlorophyll content of cotton crop substantially enhanced with application of poultry manure (10 t ha^−1^) as compared to recommended dose of synthetic fertilizers during both years of the field experimentation. Similarly, during both years of the field experimentation results of growth attributes were found satisfactory with the application of farmyard manure, compost and biochar (Table 1). Crop growth rate, peak leaf area index, and chlorophyll content of cotton crop showed an extraordinary and positive correlation with each other during both years of field study (Fig. 1).

**Table 1:** Effect of different tillage systems and organic materials on growth attributes, and chlorophyll content of cotton crop.

Treatments	Crop growth rate (g m^−2^ day^−1^)		Peak leaf area index		Chlorophyll content (SPAD index)	
	2020	2021	2020	2021	2020	2021
**Tillage systems (TS)**						
T_1_	3.62 B	3.66 B	4.03 B	4.02 B	50.6 B	50.1 B
T_2_	3.75 A	3.80 A	4.29 A	4.28 A	54.9 A	54.4 A
HSD (p ≤ 0.05)	0.105	0.108	0.061	0.065	2.94	2.93
**Organic materials (OM)**						
NPK	3.77 a	3.81 a	4.19 ab	4.18 b	53.4 b	52.9 b
FYM	3.62 b	3.67 b	4.17 b	4.16 c	51.6 c	51.1 c
BC	3.52 b	3.57 b	4.05 c	4.03 d	49.9 d	49.5 d
CM	3.62 b	3.67 b	4.19 a	4.18 b	53.6 b	53.1 b
PM	3.89 a	3.94 a	4.21 a	4.19 a	55.2 a	54.8 a
HSD (p ≤ 0.05)	0.137	0.139	0.016	0.013	1.54	1.56
TS	**	**	**	**	**	**
OM	**	**	**	**	**	**
Y	NS	NS	NS	NS	NS	NS
TS × OM	NS	NS	NS	NS	NS	NS
Y × TS	NS	NS	NS	NS	NS	NS
Y × OM	NS	NS	NS	NS	NS	NS
Y × TS × OM	NS	NS	NS	NS	NS	NS

Values sharing the same alphabet letters did not differ markedly at p ≤ 0.05 for a particular trait.

*NS* non-significant p ≤ 0.05, *T*_*1*_ conventional tillage, *T*_*2*_ conservation tillage, *FYM* farmyard manure, *BC* biochar, *CM* compost, *PM* poultry manure.

*Significant at p ≤ 0.05.

**Significant at p ≤ 0.01.

### Morphological attributes of cotton crop

The effects of different tillage systems, and organic materials were found significant (at p ≤ 0.05) on plant height, monopodial, and sympodial branches per plant of cotton crop. However, the interactive effects of different tillage systems, and organic materials, year as source of variation and its interactive with different tillage systems, and organic materials did not affect significantly plant height, monopodial, and sympodial branches per plant of cotton crop (Table 2). During both years of the field experimentation, cotton crop sown by conservation tillage system showed significantly higher plant height, monopodial, and sympodial branches per plant as compared with conventional tillage system. Furthermore, plant height, monopodial, and sympodial branches per plant of cotton crop substantially improved with application of poultry manure (10 t ha^−1^) as compared to recommended dose of synthetic fertilizers during both years of the field experimentation. The application of farmyard manure, compost and biochar also improved morphological attributes of cotton crop during both years of the field experimentation (Table 2). The plant height, monopodial, and sympodial branches per plant of cotton crop also showed an extraordinary and positive correlation with each other during both years of field study (Fig. 1).

**Table 2:** Effect of different tillage systems and organic materials on the morphological attributes of cotton crop.

Treatments	Plant height (cm)		Monopodial branches per plant		Sympodial branches per plant	
	2020	2021	2020	2021	2020	2021
**Tillage systems (TS)**						
T_1_	130 B	132 B	2.94 B	2.97 B	20.0 B	21.6 B
T_2_	142 A	144 A	3.11 A	3.13 A	25.4 A	27.0 A
HSD (p ≤ 0.05)	5.21	6.11	0.137	0.140	1.54	1.51
**Organic materials (OM)**						
NPK	139 b	141 b	3.19 a	3.22 a	23.7 a	25.6 a
FYM	131 c	133 c	2.93 b	2.97 b	21.7 b	23.6 b
BC	127 c	129 c	2.82 b	2.86 b	19.3 c	21.1 c
CM	140 b	143 b	2.94 b	2.97 b	23.8 a	25.6 a
PM	146 a	147 a	3.23 a	3.26 a	24.9 a	26.7 a
HSD (p ≤ 0.05)	4.95	5.75	0.162	0.165	1.19	1.22
TS	**	**	**	**	**	**
OM	**	**	**	**	**	**
Y	NS	NS	NS	NS	NS	NS
TS × OM	NS	NS	NS	NS	NS	NS
Y × TS	NS	NS	NS	NS	NS	NS
Y × OM	NS	NS	NS	NS	NS	NS
Y × TS × OM	NS	NS	NS	NS	NS	NS

Values sharing the same alphabet letters did not differ markedly at p ≤ 0.05 for a particular trait.

*NS* non-significant p ≤ 0.05, *T*_*1*_ conventional tillage, *T*_*2*_ conservation tillage, *FYM* farmyard manure, *BC* biochar, *CM* compost, *PM* poultry manure.

*Significant at p ≤ 0.05.

**Significant at p ≤ 0.01.

### Yield attributes of cotton crop

TB per plant, BW, SCY, and BY of cotton crop were markedly affected (at p ≤ 0.05) by different tillage systems, and organic materials. However, the interactive effects of different tillage systems, and organic materials, year as source of variation and its interactive with different tillage systems and organic materials were not found significant for the TB per plant, BW, SCY, and BY of cotton crop (Table 3). Cotton crop sown by conservation tillage system showed significantly higher TB per plant, BW, SCY, and BY of cotton crop as compared with conventional tillage system during both years of field experimentation. During both years of field experimentation, TB per plant, BW, SCY, and BY of cotton crop also substantially enhanced with application of poultry manure (10 t ha^−1^) as compared to recommended dose of synthetic fertilizers. Similarly, TB per plant, BW, SCY, and BY of cotton crop were found satisfactory with the application of farmyard manure, compost and biochar during both years of field experimentation (Table 3). TB per plant, BW, SCY, and BY of cotton crop showed an extraordinary and positive correlation with each other during both years of field study (Fig. 1).

**Table 3:** Effect of different tillage systems and organic materials on the yield attributes of cotton crop.

Treatments	Toll bolls per plant		Boll weight (g)		Seed cotton yield (kg ha^−1^)		Biological yield (kg ha^−1^)	
	2020	2021	2020	2021	2020	2021	2020	2021
**Tillage systems (TS)**								
T_1_	28.0 B	29.4 B	3.90 B	3.91 B	3409 B	3465 B	9659 B	9673 B
T_2_	31.4 A	32.7 A	4.23 A	4.24 A	3692 A	3748 A	10,341 A	10,355 A
HSD (p ≤ 0.05)	3.01	3.09	0.245	0.221	262.4	274.5	192.0	190.7
**Organic materials (OM)**								
NPK	30.3 b	31.5 b	4.12 b	4.13 b	3696 a	3751 a	10,131 a	10,145 a
FYM	28.5 c	29.7 c	3.98 c	3.99 c	3324 b	3380 b	9891 b	9905 b
BC	27.3 c	28.5 c	3.85 d	3.86 d	3174 b	3230 b	9578 c	9591 c
CM	30.5 b	31.7 b	4.13 b	4.14 b	3670 a	3726 a	10,135 a	10,149 a
PM	32.1 a	33.2 a	4.26 a	4.27 a	3888 a	3945 a	10,267 a	10,281 a
HSD (p ≤ 0.05)	1.55	1.62	0.119	0.121	226.9	238.9	145.2	144.3
TS	**	**	**	**	**	**	**	**
OM	**	**	**	**	**	**	**	**
Y	NS	NS	NS	NS	NS	NS	NS	NS
TS × OM	NS	NS	NS	NS	NS	NS	NS	NS
Y × TS	NS	NS	NS	NS	NS	NS	NS	NS
Y × OM	NS	NS	NS	NS	NS	NS	NS	NS
Y × TS × OM	NS	NS	NS	NS	NS	NS	NS	NS

Values sharing the same alphabet letters did not differ markedly at p ≤ 0.05 for a particular trait.

*NS* non-significant p ≤ 0.05, *T*_*1*_ conventional tillage, *T*_*2*_ conservation tillage, *FYM* farmyard manure, *BC* biochar, *CM* compost, *PM* poultry manure.

*Significant at p ≤ 0.05.

**Significant at p ≤ 0.01.

### Soil health indices

**Figure 2:**
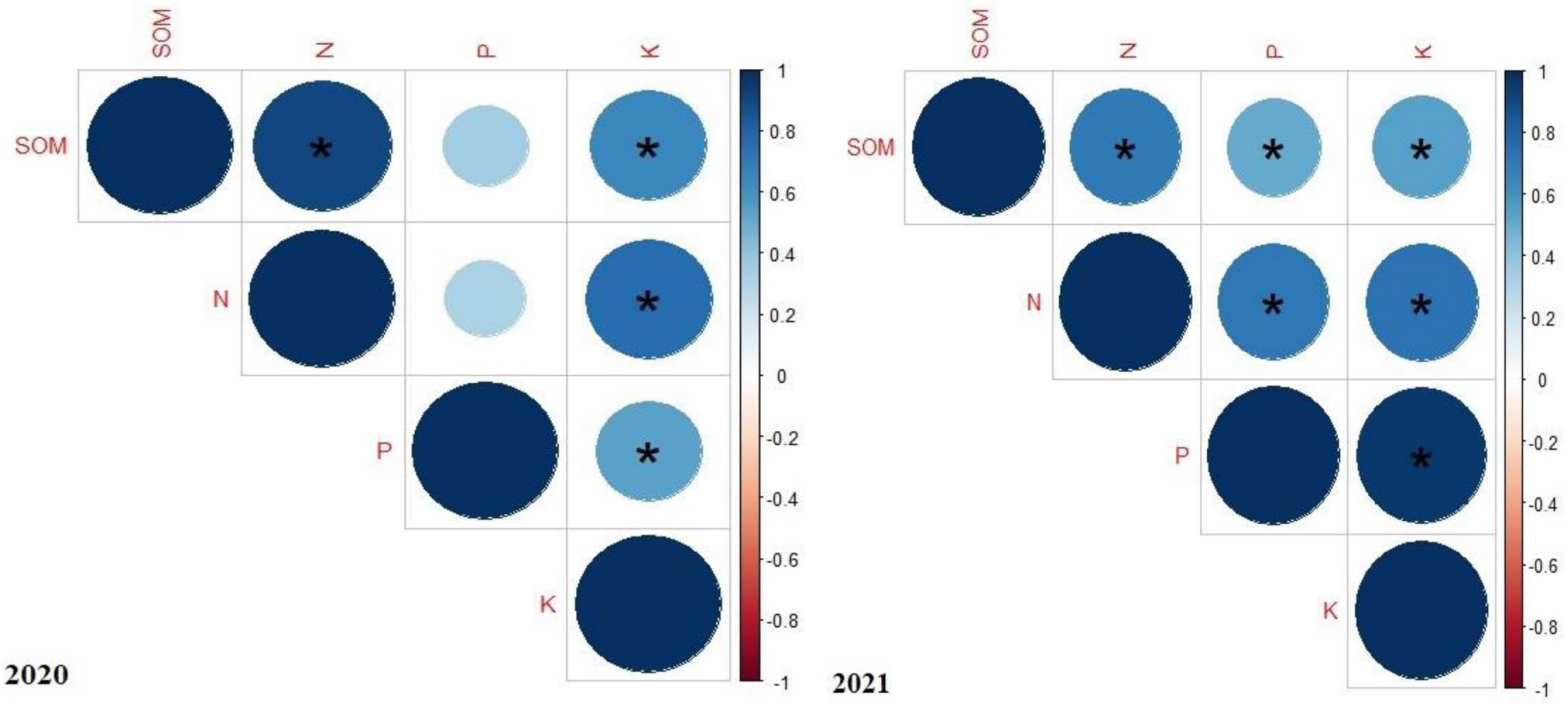
Correlation map showing the effect of different tillage systems, and organic materials on soil health indices. The areas of circles show the absolute value of corresponding correlation coefficients tested at *0.01 significance level. *SOM* soil organic matter, *N* soil available nitrogen, *P* soil available phosphorus, *K* soil available potassium. *p ≤ 0.05; **≤ 0.01; ***≤ 0.001.

The effects of different tillage systems, and organic materials were found significant (at p ≤ 0.05) on soil health indices i.e. SOM, N, P, and K. However, the interactive effects of different tillage systems, and organic materials, year as source of variation and its interactive with different tillage systems, and organic materials did not affect significantly soil health indices i.e. SOM, N, P, and K (Table 4). During both years of the field study, conservation tillage system showed significantly higher soil health indicators i.e. SOM, N, P, and K as compared with conventional tillage system. Furthermore, soil health indicators i.e. SOM, N, P, and K improved with application of poultry manure (10 t ha^−1^) as compared to recommended dose of synthetic fertilizers during both years. Likewise, the application of farmyard manure, compost and biochar also improved soil health indicators i.e. SOM, N, P, and K during years 2020, and 2021 (Table 4). Soil health indices i.e. SOM, N, P, and K also showed an extraordinary and positive correlation with each other during both years of field study (Fig. 2).

**Table 4:** Effect of different tillage systems and organic materials on the soil health indices.

Treatments	Soil organic matter (%)		Available nitrogen (mg kg^−1^)		Available phosphorus (mg kg^−1^)		Available potassium (mg kg^−1^)	
	2020	2021	2020	2021	2020	2021	2020	2021
**Tillage systems (TS)**								
T_1_	0.762 B	0.761 B	68.5 B	69.0 B	7.13 B	7.26 B	205 B	207 B
T_2_	0.809 A	0.815 A	74.3 A	74.6 A	7.29 A	7.43 A	213 A	216 A
HSD (p ≤ 0.05)	0.012	0.023	3.91	3.58	0.096	0.137	5.99	6.22
**Organic materials (OM)**								
NPK	0.791 ab	0.789 ab	72.3 b	72.8 a	7.35 ab	7.51 a	211 b	213 b
FYM	0.787 ab	0.792 ab	69.8 c	70.1 b	7.10 c	7.26 b	205 b	207 b
BC	0.763 c	0.772 b	67.6 d	68.2 b	7.12 bc	7.15 b	200 b	202 b
CM	0.791 ab	0.789 ab	72.5 b	72.8 a	7.10 c	7.26 b	206 b	207 b
PM	0.794 a	0.797 a	74.7 a	75.0 a	7.39 a	7.55 a	221 a	223 a
HSD (p ≤ 0.05)	0.022	0.021	2.10	2.53	0.236	0.162	7.80	7.62
TS	**	**	**	**	**	**	**	**
OM	**	**	**	**	**	**	**	**
Y	NS	NS	NS	NS	NS	NS	NS	NS
TS × OM	NS	NS	NS	NS	NS	NS	NS	NS
Y × TS	NS	NS	NS	NS	NS	NS	NS	NS
Y × OM	NS	NS	NS	NS	NS	NS	NS	NS
Y × TS × OM	NS	NS	NS	NS	NS	NS	NS	NS

Values sharing the same alphabet letters did not differ markedly at p ≤ 0.05 for a particular trait.

*NS* non-significant p ≤ 0.05, *T*_*1*_ conventional tillage, *T*_*2*_ conservation tillage, *FYM* farmyard manure, *BC* biochar, *CM* compost, *PM* poultry manure.

*Significant at p ≤ 0.05.

**Significant at p ≤ 0.01.

## Discussion

After two years of field experimentation, we found that studied different tillage systems (T_1_, and T_2_) and organic materials (CM, BC, PM, and FYM) significantly improved the growth, morphological, and yield traits of cotton during 2020, and 2021. Cotton crop sown under T_2_ showed significantly higher crop growth rate, peak leaf area index, and chlorophyll content as compared with T_1_ during both years of the field experimentation (Table 1). The present significant differences in the crop growth rate, peak leaf area index, and chlorophyll content of cotton crop was also previously reported in other studies^40^. These results were correlated with the more soil organic carbon, moisture retention and hydraulic conductivity, nutrients cycling, and soil organic matter under conservation tillage system^41,42^. Similarly, T_2_ also showed rising trend for PH, MB, and SB per plant of cotton as compared to T_1_ during both years of the field experimentation (Table 1). These results are associated with the fact that decomposition of organic amendments slowly releases the nutrients, and the losses of nutrients are reduced under minimum tillage due to improved soil structure^35^ which might be a reason behind improved growth and yield traits of cotton. Additionally, higher growth, morphological, and yield attributes were associated with positive effect of reduced tillage practices and residue retention on soil properties such as organic matter, nutrients availability, especially total nitrogen and decreased soil bulk density and improved soil porosity^42–44^. Improved soil porosity leads to proper root growth and development and ultimately optimum growth and yield traits of cotton crop as observed in the current study.

Furthermore, crop growth rate, peak leaf area index, and chlorophyll content of cotton crop substantially improved with application of PM (10 t ha^−1^) as compared to recommended dose of synthetic fertilizers during both years of the field experimentation. Poultry manure (PM) consists of macro and micronutrients^6,45^, readily available for plants growth that might be a possible reason behind improved growth traits of cotton. Higher growth traits of cotton crop might be due to higher chlorophyll content production recorded in the same treatment during both growing seasons (Table 1). Many other studies presented improvement in the growth and physiological traits with application of poultry manure as an organic fertilizer source^46,47^ which was attributed to its vital role in moisture conservation, improving soil porosity and bulk density These were also closely associated with the fact that organic materials decompose and discharge different nutrients slowly^48,49^ which may enhance the growth attributes of the crop under amended experimental units. Furthermore, plant height, monopodial, and sympodial branches per plant of cotton crop were improved with application of PM (10 t ha^−1^) in comparison to recommended dose of synthetic fertilizers during both years of the field experimentation (Table 2). These results were associated with the fact that decomposition of organic amendments such as poultry manure slowly releases the nutrients, and reduces the nutrients losses^50^. In contrary, nutrients applied through synthetic fertilizers especially nitrogenous fertilizers are easily leached down that led to low fertilizer use efficiency and decreased crop traits. Moreover, TB per plant, BW, SCY, and BY of cotton crop also showed increasing trend with the application of PM (10 t ha^−1^) as compared to recommended dose of synthetic fertilizers (Table 2). These results are in agreement to other studies which have supported that poultry manure increases the growth and yield attributes of cotton crop^6^. Higher yield components were correlated with the fact of higher availability of macronutrients of N, P, and K through poultry manure throughout the growing period and reduced losses of nutrients under field conditions^6,51^. Furthermore, poultry manure reduces the soil pH due to acidic in nature that led to higher growth, morphological and yield traits of cotton in the current study. Higher seed cotton yield of cotton crop might be due to highest number of total bolls per plant and boll weight recorded in the same treatment during both growing seasons (Table 3). There were also positive results of studied growth, morphological, and yield traits of cotton with the application of FYM, CM and BC as shown in Tables 1, 2 and 3. These outcomes had proved earlier by several studies^6,7,52^. Moreover, previous studies have also shown higher growth and yield traits of wheat^53^ and maize^54,55^ with the application of poultry manure as compared to farmyard manure and recommended dose of synthetic fertilizers.

Soil health indices i.e. SOM, N, P, and K were improved significantly due to positive effects of different tillage systems and organic amendments during both years 2020, and 2021 (Table 4). Conservation tillage system showed the highest SOM, N, P, and K as compared to conventional tillage system during both years of field experimentation (Table 3). The higher SOM, N, P, and K in T_2_ (Table 4) may be attributed to declined soil and water losses through erosion and leaching, and more soil nutrients and organic carbon accumulation^56–58^. This also occurs due to the environment more favorable for decomposition under T_2_^34^. Our results suggest that T_2_ can enhance soil health indices by reducing the losses of soil and water that led to reduced losses of nutrients. Moreover, these results have proved by one field trail which showed similar trend in SOM, and N of soils after nine years of T_2_ system^59^. Conservation tillage system increases the soil porosity, reduces the soil bulk density and hence more nutrients holding capacity that might be reason behind the more SOM, N, P, and K in T_2_.

In our study, application of PM (10 t ha^−1^) showed highest SOM, N, P, and K (Table 4). Higher SOM and N were recorded with the application of PM (10 t ha^−1^) which was attributed to more nutrients addition on decomposition of poultry manure^6^. Similar studies have proved that soil amendment of poultry manure improves the soil organic matter and soil available nitrogen^6,60^. Poultry manure increases the soil porosity and reduces the nutrients losses especially mobile nutrients such as nitrogen which might be reason behind increased nitrogen availability in the soil. Furthermore, higher P, and K was also recorded with the application of PM (10 t ha^−1^) which was attributed to nutrients addition on decomposition of poultry manure^6^. Being an acidic in nature, application of poultry manure leads to the reduced soil pH which solubilized the phosphate and increased soil phosphorus and potassium as observed in the current study. Many other studies have confirmed these results and showed positive increasing trend in soil available phosphorus and potassium with the application of poultry manure^6,51,60^.

## Materials and methods

### Study site

**Figure 3:**
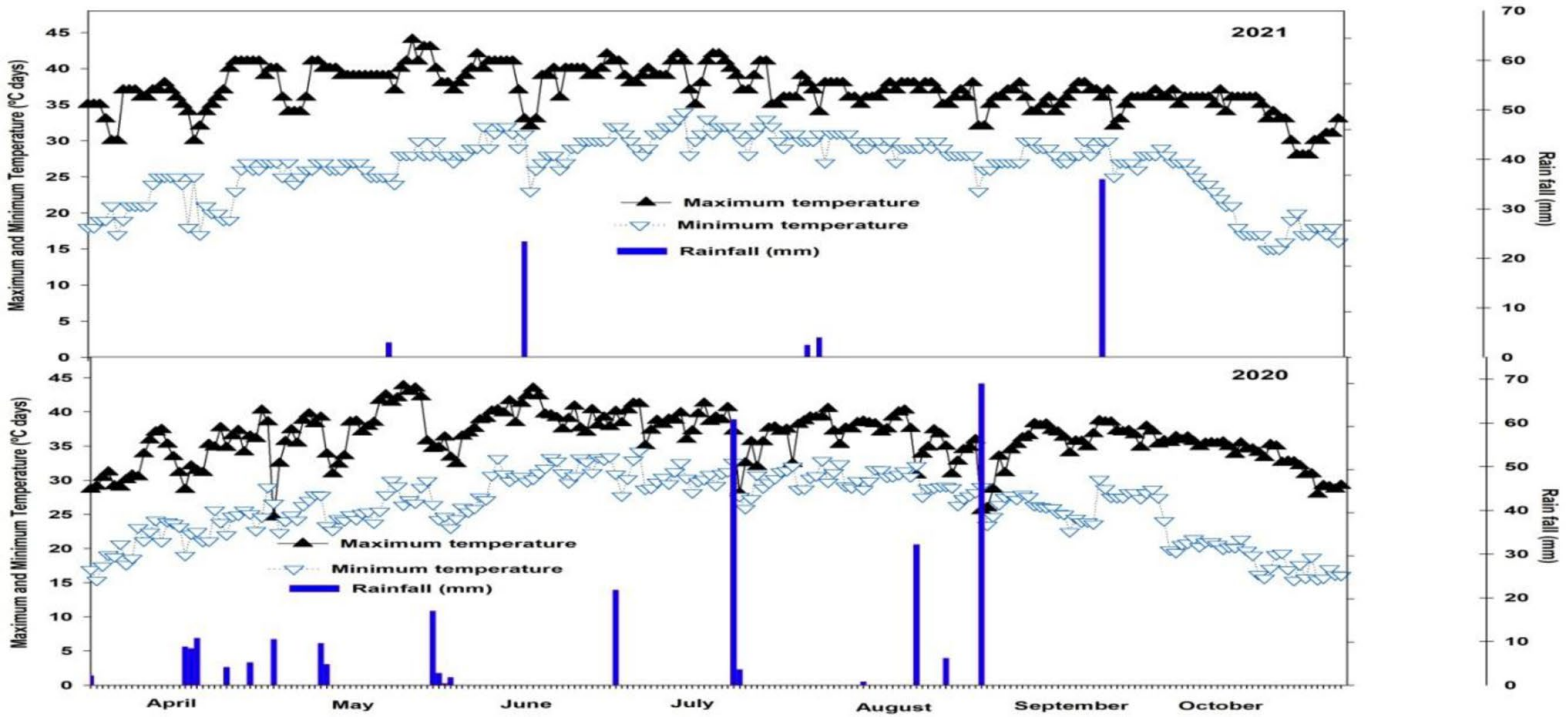
Weather data during the experimental period of cotton crop during both years 2020, and 2021.

A cotton field experiment was conducted in consecutive two growing seasons during last week of April–second week of October 2020 and 2021 at MNS University of Agriculture, Multan (30° 15 N, 71° 53 E). The area is located in the Southern region of Punjab, Pakistan. The climate of experimental area is semi-arid. Weather data of field study of two growing seasons 2020, and 2021 was collected from Weather Station installed at MNS University of Agriculture, Multan, is presented in Fig. 3. The top layer soil is 30% clay (< 0.002 mm), 30% silt (0.002–0.05 mm) and 40% sand (0.05–2.0 mm). Additionally, analysis of top layer soil showed 8.20, 70.5 mg kg^−1^, 0.78%, 7.50 mg kg^−1^, and 210 mg kg^−1^, pH, soil available nitrogen, phosphorus, potassium, and soil organic matter, respectively.

### Experimental design and treatments

Proceeding to this field experiment, potato crop was grown in 2019 on the same land. After the harvesting of potato crop, biochar, compost, farmyard and poultry manures (organic materials) were spread out on the surface based on experimental treatments to all corresponding experimental units, about one month prior to the sowing of cotton crop. Then, the land was prepared on 15 March 2020 and 17 March 2021 for the first and second growing seasons, respectively. Soil was plowed to ensure the thoroughly mixing of organic materials after the incorporation.

The current field experiment was conducted in a split plot design with three replications. The experimental treatments were comprised of different tillage systems (main plots): conventional tillage (T_1_), and conservation tillage system (T_2_), and organic materials (sub-plots): NPK (control or recommended dose of fertilizers; 160:90:60 kg ha^−1^NPK), biochar (BC), farmyard manure (FYM), compost (CM), and poultry manure (PM) which are presented in detail in Table 5. Conventional tillage (T_1_) included one ploughing, one harrowing, two hoeing and two hand weeding. It is a widely used by the farmers worldwide. Conservation tillage system (T_2_) included one ploughing, and two hand weeding. It is an advance and sustainable tillage system that is also a major component of conservation agriculture. Studied organic materials were spread to all the corresponding experimental units one month before the execution of T_1_ and T_2_. The studied treatments were allocated in the same experimental units during both years of field experimentation. The size of experimental unit was 10 m × 9.0 m. Seeds were sown on April 25, 2020, and April 27, 2021 by hand drilling.

**Table 5:** Preparation method, nutritional composition, and source of biochar, compost, poultry and farmyard manure.

Organic material	Preparation	Composition	Source
Biochar	Under slow pyrolysis with limited presence of oxygen in Kon-Tiki Flame Curtin Pyrolysis Biochar Kiln at 400° temperature^6,7^ The pyrolysis reaction in the Kiln was quenched with the application of water. Produced biochar was air dried and used on dry weight basis	The nutritional composition of biochar was consisted of 36.5% C, 1.36% N, 0.14% P, 1.97% K, 95 g kg^−1^ Ca, 5.16 g kg^−1^ Mg, 25.3 g kg^−1^ Fe and 586 mg kg^−1^ Mn	Cotton sticks were collected from Farmer field
Compost	Compost was made from 50% green waste (25% dry matter), 35% chopped wood (60% dry matter), and 15% soil with woody debris	The nutritional composition of biochar was consisted of 36.9% TOC, 2.36% N, 1.49% P, and 1.77% K	Layyah Sugar Mill
Poultry manure	Poultry manure (PM) was spread and incorporated into the soil	The nutritional composition of poultry manure was consisted of 1.12% N, 0.12 ppm P, 0.07 ppm K, 230 ppm Fe and 0.47 ppm Mn, 50.5 ppm Cu, 350 ppm Zn and 11.7 ppm B	Faisal Poultry Shed
Farmyard manure	Farmyard manure (FYM) was spread and incorporated into the soil	Nutritional composition of FYM was consisted of 1.10% N, 0.08 ppm P, 0.06 ppm K, 430 ppm Fe and 0.45 ppm Mn, 3.00 ppm Cu, 158 ppm Zn and 10.6 ppm B	Hussain and Sons Livestock Farm

### Sampling and measurements

#### Soil health indices

Soil samples to the depth of 30 cm from each experimental unit were collected with the help of soil augar and were analyzed at cotton harvest during both years of field experiments. To ensure uniform size, these were sieved (2-mm mesh) and sun-dried. Soil organic matter was determined using Walkley and Black^61^. For measuring soil organic matter, 1.0 g of grinded soil sample and 10 mL of 1 N K_2_Cr_2_O_7_ and H_2_SO_4_ was transferred to 250 mL Erlenmeyer, mixed uniformly and kept for 30 min. Then, we added 3 mL of H_3_PO_4_, 50 mL of DI water, and 0.5 mL of 1.0% defenilamina indicator in the mixture. Using 1 N FeSO_4_ solution, mixture was titrated slowly until it showed green color point and recording of soil organic matter was noted. Soil available nitrogen was estimated using alkaline permanganate technique^62^. In this technique, 20 g air dried soil, distilled water (20 mL), 0.320% KMnO_4_ solution (100 mL), 2.50% NaOH solution (100 mL) were taken in 800 mL Kjeldahl digestion flask. From Kjeldahl digestion flask, 75 mL distillate was taken and 25 mL of boric acid was added and Bromocresol green and Methyl red as indicators were used. The absorbed ammonia was titrated using 0.05 N H_2_SO_4_ to determine available nitrogen content in the soil sample. Soil available phosphorus was determined using sodium bicarbonate^63^. In this procedure, 2.0 g air-dried soil was taken into 250 mL digestion tube and 30 mL of 60% HClO_3_ with some pumice-boiling granules. On block-digester, tubes rack were placed and lightly heated at 100 °C until 180 °C and soil samples were digested until dense white fumes of acid appeared and kept for 40 min to cold it. It was filtered using Whatman No. 1 filter paper. We took 5 mL of filtrate, ammonium-vanadomolybdate (10 mL) reagent and DI H_2_O in 50 mL flask. Spectrophotometer was run at 420 nm wavelength and the concentration of phosphorus in the digested soil samples was determined. Soil available potassium was estimated using ammonium acetate method^64^. In this method, 10 g air-dry soil and 50 mL of 1 N NH_4_OAc solution were taken in 250-mL flask and mixed uniformly on shaker at 200–300 rpm for half hour, and filtered using Whatman No. 1 filter paper. Flame Photometer run at 767 nm wavelength and concentration of potassium was estimated.

### Growth, morphological, and yield attributes

Different growth, morphological, and yield attributes of the cotton were studied and data was recorded for evaluation. Crop growth rate (CGR) of tagged plants at 150 days after sowing (DAS) was estimated by using method of Watson^65^. Chlorophyll content (CHC) of the tagged plants was determined with the help of a chlorophyll meter (SPAD-502; Minolta, Tokyo, Japan). Peak leaf area index (LAI) was estimated via the method of Sestak, Catsky, and Jarvis^66^. Leaf area of samples (5 g cotton leaves) was calculated with the help of leaf area meter. To estimate the leaf area index, land area (m^2^ plant^−1^) was divided by the leaf area (m^2^ plant^−1^). At maturity, randomly ten plants were tagged and plant height (PH) from base to tip of the plant’s main stem was recorded with the help of measuring tape and its mean was calculated. Similarly, sympodial (SB), monopodial branches (MB), and total bolls (TB) of tagged ten plants were counted and their mean was calculated at 150 DAS. Boll weight (BW) of selected ten mature and effective bolls was calculated and its mean was taken out. Weight of seed cotton yield (SCY) recorded from already picked 10 bolls was added into the weight of SCY recorded from the net plot and was converted as SCY (kg ha^−1^). To calculate biological yield (BY), the plants were harvested from one meter square area and were separated into leaves, stem, and reproductive parts and oven-dried at 65–70 °C to a constant weight. The recorded dry weight of samples was converted into BY (kg ha^−1^).

### Statistical analysis

Experimental data was analyzed statistically with the help of analysis of variance technique to find out the influence of different tillage systems and organic materials on growth, morphological, and yield traits of cotton, and soil health indices under an arid environment. Additionally, Tukey’s Honest Significant Difference (HSD) was applied to find out the significant differences between treatments means at p ≤ 0.05^67^.

### Ethics approval and consent to participate

We all declare that paper reporting studies do not involve any human participation, human data, or human tissues. So, it is not applicable.

### Consent for publication

Our paper does not contain data from any individual person. So, it is not applicable.

### Plant guidelines

All the plant experiments were in compliance with relevant institutional, national, and international guidelines and legislations.

## Conclusion

Results of 2 years field experimental trials indicated that different tillage systems and organic materials significantly affected the growth, morphological, and yield traits of cotton and soil health indices. Higher growth, morphological, and yield traits of cotton, and soil health indices were recorded under T_2_ as compared to T_1_ system. Similarly, PM (10 t ha^−1^) showed higher growth, morphological, and yield attributes of cotton and soil health indices as compared to FYM (20 t ha^−1^), CM (10 t ha^−1^), and BC (7 t ha^−1^). In conclusion, conservation tillage system, and application of 10 t ha^−1^ poultry manure might be a pragmatic choice for improving cotton productivity, and soil health indices under arid climatic conditions. These findings are recommended for the farmers to improve the cotton production and sustain the soil health indices. Further studies may explore with processed based dynamic simulation models to see the impact of these findings under future climate and assess the potential of these strategies to sustain the soil health indices and ensure sustainable cotton production under future climate change scenarios.

## Data Availability

Datasets and codes used and/or analyzed during the present study are available from corresponding author on reasonable request.
